# Supplementary material for: Assessing Trypanosoma cruzi Parasite Diversity through Comparative Genomics: Implications for Disease Epidemiology and Diagnostics
Source: Pathogens. 2021 Feb 16;10(2):212. doi: 10.3390/pathogens10020212 (PMC7919814; doi:10.3390/pathogens10020212)
Supplement: Supplementary file 1 [file pathogens-10-00212-s001.pdf]

Table S1. Markers used to assess *T. cruzi* diversity.

**Marker Name**	**Accession No.**	**Size (bp)**
18s	AY785564	2260
24s	AY367115	118
HP1	XM_804213.1	1206
HP2	XM_804586.1	1170
Ferric Reductase Transmembrane Protein	XM_806472.1	723
HP4	XM_806040.1	1455
HP5	XM_812200.1	1368
Endomembrane Protein	XM_801081.1	1797
Beta-adaptin	XM_815241.1	2898
HP8	XM_809640.1	954
HP9	XM_816825.1	1359
HP10	XM_811962.1	1935
Protein kinase	XM_811220.1	834
HP12	XM_803751.1	1047
HP14	XM_803821.1	1218
HP15	XM_810049.1	924
Thiol-dependent reductase 1	XM_802287.1	1338
Flagellum-adhesion glycoprotein	XM_800084.1	1746
HP18	XM_810193	708
HP19	XM_816163.1	1365
HP20	XM_802518.1	954
HP21	XM_804543.1	1644
HP22	XM_800573.1	1368
HP23	XM_802177.1	795
HP24	XM_815790.1	1908
HP25	XM_816871.1	2112

HP26	XM_808681.1	552
HP27	XM_810645.1	795
HSP70	KC959991.1	1380
MSH2	AY092834.1	829
SAT	HQ859509.1	805
TcSD5D	XM_799062	837

Accession numbers correspond to marker sequence in the CL-Brener genome, except for SAT, HSP70, and 18s, which correspond to marker sequence in the Esmeraldo genome and 24s, which corresponds to marker sequence in the CanIII genome. HP: hypothetical protein

Table S2. Mini-exon copy number in *T. cruzi* genomes

Strain Name - DTU	# of Sequences*
231 - TcIII	4
Arequipa - TcI	636
CanIII - TcIV	13
CGI14 - TcI	150
CLBrener - TcVI	295
Colombiana - TcI	653
Corpus Christi - TcI	286
Esmeraldo - TcII	20
FcHcI1 - TcI	217
H1 Panama -TcVI	154
H2 - TcI	151
H3 - TcI	142
H5 --TcI	122
H6 - TcI	122
H7 - TcI	123
H9 - TcI	104
H12 - TcI	136
H14 - TcI	125
H15 - TcI	128
Jose - TcI	424
JRc14 - TcI	52
M6241 - TcIII	13
TBM3479B1 - TcI	26
TBM3519W1 - TcI	122
Bug2148 - TcI	173
TD23 - TcI	167
TD25 - TcI	151
TMB3406B1 - TcI	148

Tula cl2 - TcVI	21
V1 - TcI	169
V2 -TcI	126
V3 - TcI	167
X10462 - TcI	170
X12422 - TcI	131
Y - TcII	233
G - TcI	1
S11 - TcII	4
S15 - TcII	7
TCC - TcVI	225
Ycl4 - TcII	1
9280cl2 - TcV	214
H4 -TcI	160
s154a - TcI	2
H1Yuc - TcI	133
Dm28c - TcI	746
S162a - TcII	6
S23b - TcII	7
S44 - TcII	4
S92a - TcII	4

*Low copy number was frequently corresponding to incomplete mini-exon sequences.

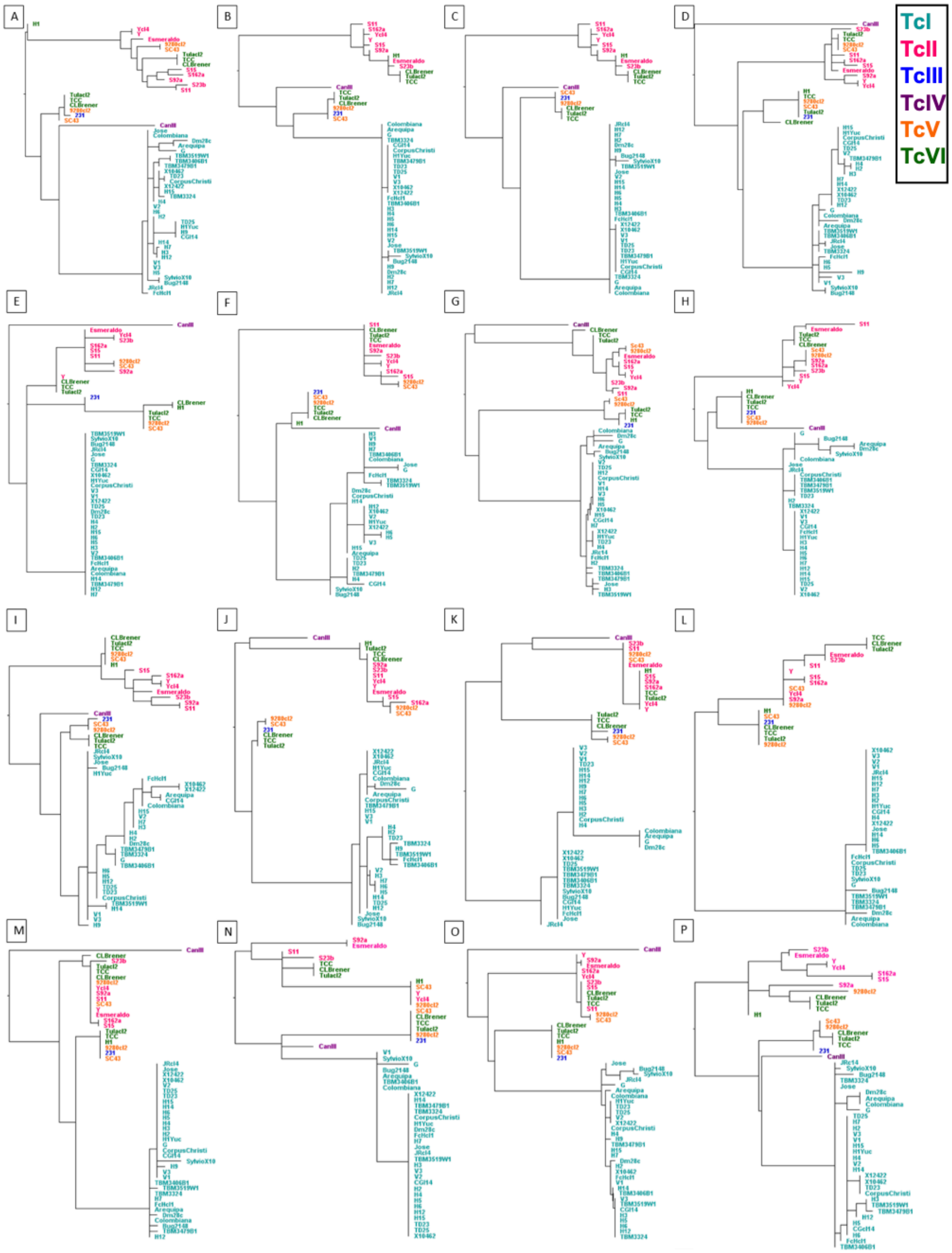
A
B
C
D
E
F
G
H
I
J
K
L
M
N
O
P
TcI
TcII
TcIII
TcIV
TcV
TcVI

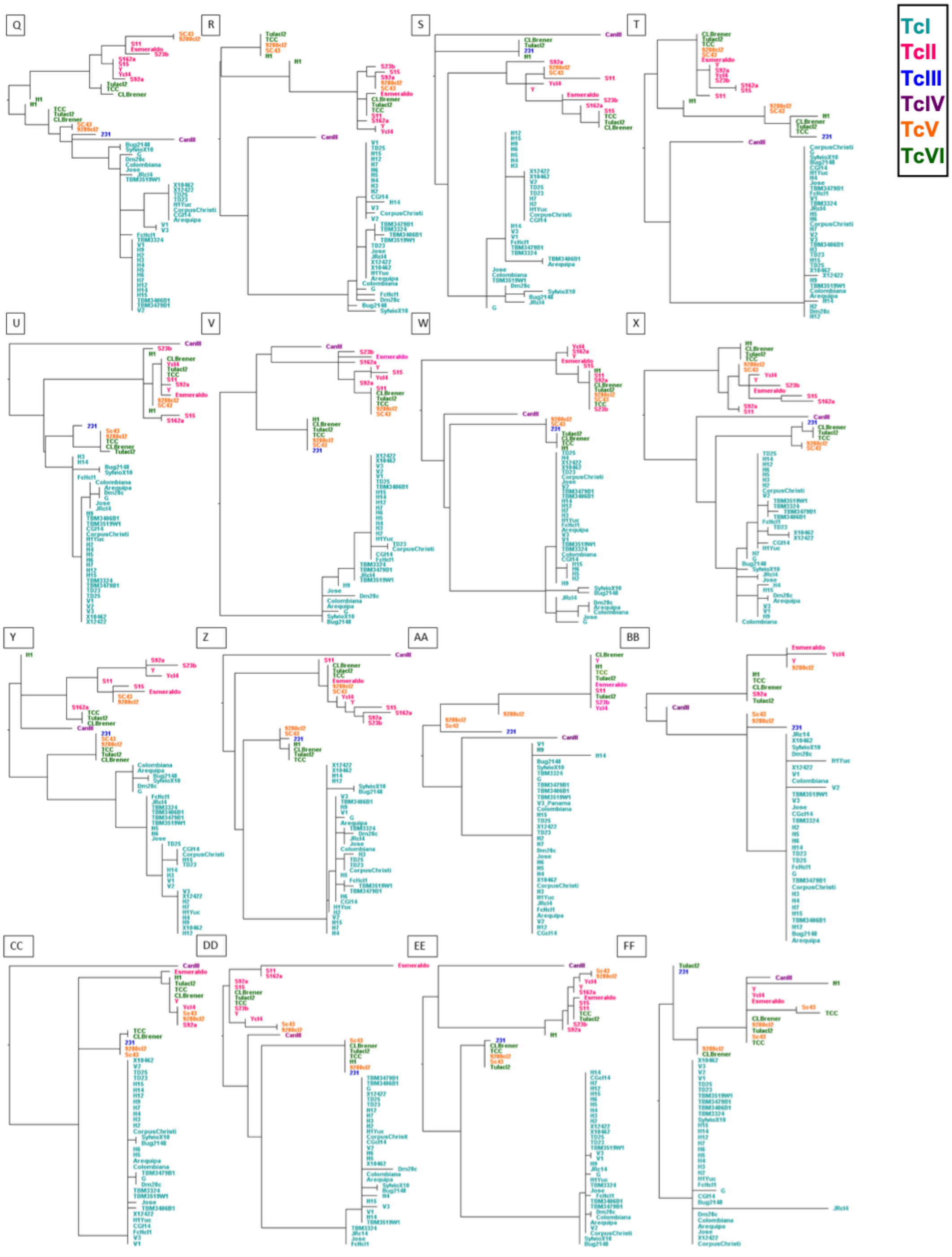
Q
R
S
T
U
V
W
X
Y
Z
AA
BB
CC
DD
EE
FF
TcI
TcII
TcIII
TcIV
TcV
TcVI

Figure S1. Phylogenetic trees constructed for individual markers.

(A) HP1, **(B)** HP2, **(C)** Ferric Reductase Transmembrane Protein, **(D)** Hypothetical protein HP4, **(E)** HP5, **(F)** Endomembrane Protein, **(G)** Beta-adaptin, **(H)** HP8, **(I)** HP9, **(J)** HP10, **(K)** Protein kinase, **(L)** HP12, **(M)** HP14, **(N)** HP15, **(O)** Thiol-dependent reductase 1, **(P)** Flagellum-adhesion glycoprotein, **(Q)** HP18, **(R)** HP19, **(S)** HP20, **(T)** HP21, **(U)** HP22, **(V)** HP23, **(W)** HP24, **(X)** HP25, **(Y)** HP26, **(Z)** HP27, **(AA)** 18S RNA, **(BB)** 24S RNA, **(CC)** SAT, **(DD)** MSH2, **(EE)** TcSD5D, **(FF)** HSP70.

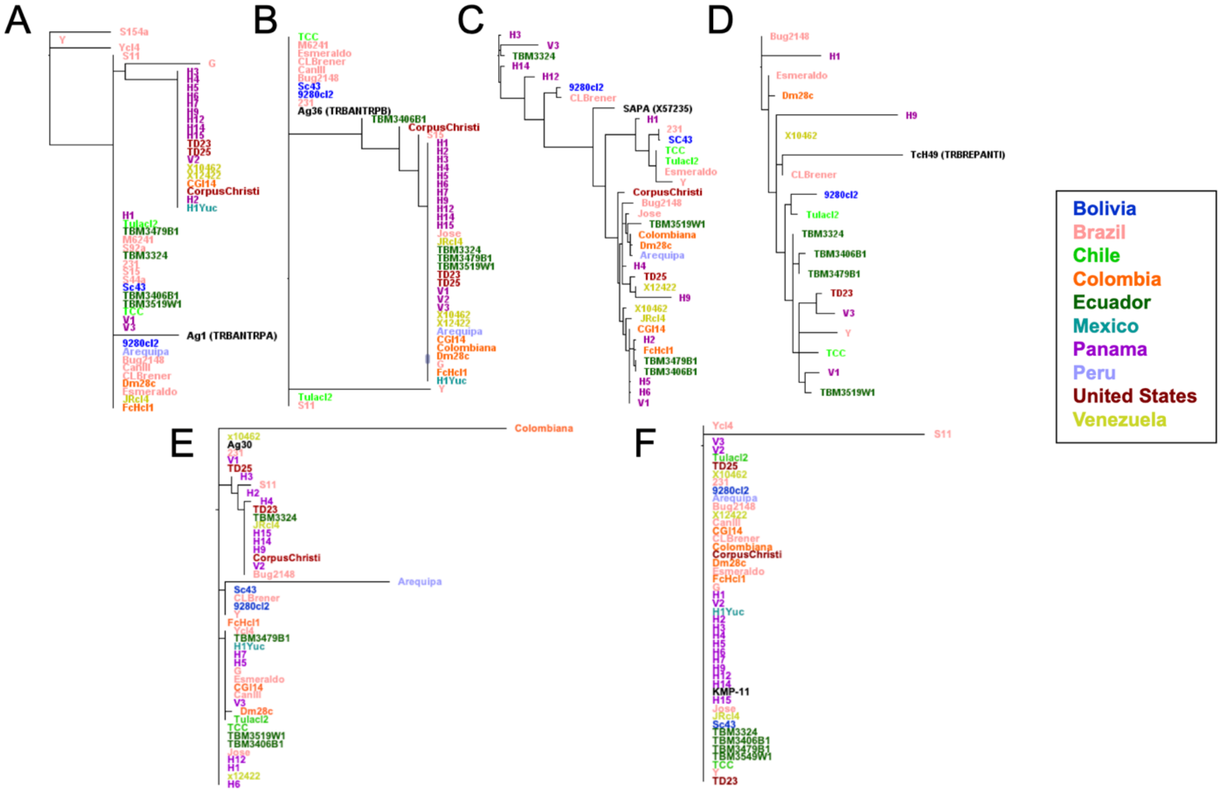

Figure S2. Phylogenetic trees for antigen sequences used in current serologic diagnostics.

(A) Ag1, **(B)** Ag36, **(C)** SAPA full length antigen, **(D)** TcH49, **(E)** Ag30, **(F)** KMP-11. Sequences in black correspond to reference antigen sequence, and the country of origin of each strain is color-coded as indicated.
